# Approaches to Improve Chemically Defined Synthetic Peptide Vaccines

**DOI:** 10.3389/fimmu.2018.00884

**Published:** 2018-04-26

**Authors:** Brett J. Hos, Elena Tondini, Sander I. van Kasteren, Ferry Ossendorp

**Affiliations:** ^1^Department of Immunohematology and Blood Transfusion, Leiden University Medical Center, Leiden, Netherlands; ^2^Leiden Institute of Chemistry, The Institute for Chemical Immunology, Leiden University, Leiden, Netherlands

**Keywords:** peptide vaccination, click chemistry, antigen presentation, intracellular processing, targeted vaccination, tumor immunology, toll-like receptors, bioorthogonal

## Abstract

Progress made in peptide-based vaccinations to induce T-cell-dependent immune responses against cancer has invigorated the search for optimal vaccine modalities. Design of new vaccine strategies intrinsically depends on the knowledge of antigen handling and optimal epitope presentation in both major histocompatibility complex class I and -II molecules by professional antigen-presenting cells to induce robust CD8 and CD4 T-cell responses. Although there is a steady increase in the understanding of the underlying mechanisms that bridges innate and adaptive immunology, many questions remain to be answered. Moreover, we are in the early stage of exploiting this knowledge to clinical advantage. Several adaptations of peptide-based vaccines like peptide-adjuvant conjugates have been explored and showed beneficial outcomes in preclinical models; but in the clinical trials conducted so far, mixed results were obtained. A major limiting factor to unravel antigen handling mechanistically is the lack of tools to efficiently track peptide vaccines at the molecular and (sub)cellular level. In this mini-review, we will discuss options to develop molecular tools for improving, as well as studying, peptide-based vaccines.

## Introduction

Recent breakthroughs in immunotherapy of cancer have unveiled that clinical responses correlate with activation and expansion of tumor-specific T lymphocytes that mostly target mutation-based neo-antigens (1–6). Alongside, induction of tumor-specific T-cell responses has been achieved with well-defined peptide-based vaccines in preclinical and clinical settings (7–10). This indicates that therapeutic vaccination with well-defined synthetically produced neo-antigenic peptides is a viable strategy.

Immunogenicity of synthetic peptide-based vaccines can be significantly influenced by the mode of delivery (11–17). For example, efficiency of cytotoxic T-cell activation and anti-tumor immune responses is improved when peptides are encapsulated in liposomes or covalently conjugated to adjuvants (18, 19). Such modifications will allow optimal uptake of antigenic peptides from the vaccination site by specialized antigen-presenting cells (APCs) with efficient proteolytic processing for major histocompatibility complex (MHC) class I and class II presentation to CD8+ cytotoxic- or CD4+ helper-T cells, respectively (20). However, the development of optimal peptide delivery modalities is non-trivial and largely remains a process of trial-and-error based on time-intensive and indirect read-out systems. Most of what is known about *in vivo* processing routes of peptides is based on murine models and little data are available in humans. Additionally, the sequence and amino acid composition may alter the physical properties and immunological behavior of individual peptides. Therefore, mechanisms of intracellular routing and processing of administered peptides in APC require in depth examination.

The processing machineries for peptide loading in MHC class I or -II for presentation is characterized by distinct protease systems. While MHC class I processing pathways involves cytosolic proteasomes, peptidases and ER-resident trimming aminopeptidases, MHC class II peptide production takes place in endolysomal compartments involving cathepsin-like proteases (21, 22). The cell biology of antigen presentation and cross-presentation, a specialized mechanism to process exogenous engulfed protein antigen for MHC class I, by dendritic cells (DCs) is not fully elucidated in detail but is crucial knowledge for optimal vaccine design (20, 22). Improving our knowledge on vaccine behavior is therefore essential for rationally designing peptide-based vaccines. However, tracking of vaccine components through the developing stages of an immune response remains difficult with present day techniques. Bulky labeling groups that are used to visualize peptides affect their physiochemical properties, which likely alters the way a peptide is internalized, processed, and presented. It is therefore vital to apply detection strategies that minimally impact the processing of peptides.

This mini-review encompasses our current knowledge of peptide-based vaccine modalities, the possibilities to properly target them through defined alterations, novel options for rational design, and the development of (bio)chemical visualization tools to improve our understanding of peptide-based vaccine behavior *in vivo*.

## Peptide Vaccination History

Peptide vaccination is based on the biological concept that induction of a T-cell response relies on the specificity of the T-cell receptor to recognize a presented oligopeptide-epitope. This epitope corresponds to only a fraction of the entire protein (polypeptide) antigen. Therefore, to initiate a T-cell response against a specific protein, a vaccine essentially needs to include only the minimal immunogenic peptide sequence which can be produced synthetically.

Vaccination with minimal epitopes in form of synthetic peptides was shown to raise antigen-specific T-cell responses (23) and represented an exciting step forward in modern vaccination biology. Immunogenicity studies in preclinical models showed effective induction of T-cell responses and the potential for its application in cancer immunotherapy was recognized. However, clinical translation of this concept did not lead to the results anticipated by the first studies (24–29). As an example, vaccination with the immunogenic peptide of the differentiation antigen gp100 for the treatment of melanoma, failed to elicit sufficiently effective T-cell responses in several clinical trials, even when relatively high numbers of antigen-specific cells were detected (30–32).

Comprehensive *in vivo* studies have revealed that, rather than the exact epitope, peptides consisting of a termini-extended sequence (long peptides) promotes higher quality T-cell responses (33). In fact, exact epitopes can directly bind on MHC class I molecules present on the surface of any somatic cell, most of which are non-professional APCs, which causes suboptimal T-cell priming. On the other hand, long peptides are processing-dependent and can be presented only by professional APC, which are specialized and equipped for engulfing, processing, and presenting the antigenic peptides coinciding with optimal T-cell co-stimulation (34, 35).

The first peptide vaccination studies in humans were carried out with long peptides derived from self-antigens mucin and HER-2/neu, and mutated K-RAS. These studies reported safety of synthetic peptide administration and an observation of tumor- or antigen-specific T-cell responses (36–39). These clinical studies provided the basis for the use of long peptides as a strategy to design more efficacious vaccines for cancer treatment. In a study conducted in an HPV-induced preclinical model, vaccination with a 35 amino acid long synthetic peptide covering a CTL and a T helper epitope of the HPV16 E7 protein, improved T-cell responses compared with vaccination with minimal epitopes and controlled tumor growth (40). The use of long peptides bolstered priming by professional APCs that resulted in higher T-cell expansion, memory formation, and markedly improved efficacy. This paved the way for clinical testing of a mixture of overlapping peptides of 32–35 amino acids covering the sequence of the E6 and E7 HPV16 proteins for the treatment of HPV-associated gynecological tumors (10, 41).

Synthetic peptide vaccination also holds high potential for the novel field of cancer vaccination against mutation-derived neo-antigens. The ambition of raising an immune response against tumor-specific mutated proteins by vaccination represents an exciting challenge that has animated cancer therapeutic research over the last few years. Efforts needed in determining the MHC-restricted epitopes may be bypassed by designing a peptide that spans the amino acid sequence on either side of the mutation. Interestingly, this concept has been successfully applied in a recent phase I study on melanoma patients (9). In this study, six patients were vaccinated with 13–20 different peptides of 15–30 amino acids designed to target an equal amount of patient-specific somatic mutations of the sequenced tumor. All patients exhibited enhanced neo-antigen-specific T-cell populations after peptide vaccination and displayed objective clinical responses, even though two patients required a supplemental treatment with anti-PD1 immuno-modulatory antibody to reach complete tumor regression. In perspective, the use of multiple long peptides for vaccination may be complicated, as the behavior of different amino acids sequences, in terms of physico-chemical properties, solubility, and bio-distribution may differ.

Concurrently, a similar approach has been developed by encoding selected patient-specific epitopes in RNA molecules (8), as the window of physico-chemical properties is smaller for these oligomers than for peptides. Also this RNA-based vaccination was able to induce a personalized tumor-specific T-cell response with clinical benefits. Both studies represent an important proof of concept for the field of neo-antigen vaccination and stimulate research to progress toward the most effective vaccination approach.

## Synthetic Peptides: Versatile Vaccine Antigens

One advantage in the use of synthetic peptides as vaccines from both an immunological and a chemical point of view is their versatility. Immunologically, peptide vaccines induce better T-cell responses compared with full protein vaccines (42, 43). In fact, peptides are more efficiently endocytosed, processed, and presented on MHC molecules compared with full proteins. Other, less understood, aspects of antigen handling by APC, indicate that antigen cross-presentation—on a mole-for-mole ratio—is more optimal for peptides than protein. This is perhaps due to efficient translocation of peptides into the cytoplasm from endosomes (44).

On the other hand, peptides are chemically easier to produce than protein antigens as they do not necessitate folding into a tertiary structure. The high throughput and parallel production set-ups for synthetic peptides allows that several variations in the linear sequence can be made to refine vaccine formulation. Collateral problems such as induction of tolerance or suboptimal priming (45, 46) can potentially be circumvented by conjugation to “adjuvant” molecules that allow targeting of APCs and contribute to adequate immune-stimulation.

A feasible strategy to improve APC targeting of synthetic peptides and at the same time deliver the right signals is to integrate ligands of pattern-recognition receptors (PRRs), such as C-type lectin- (CLR), toll-like- (TLR), and NOD-like-receptors. These receptors are highly expressed by professional APCs and are essential for pathogen sensing and immune-stimulation. Different ligands have been identified for these receptors which can be employed for targeting and immune-stimulation. This approach can also modulate the internalization routing of endocytosed antigen (47). For example, the CLR-specific mannosylation of long peptides canalized intracellular trafficking toward early endosomal low-degradative compartments rather than lysosomes for degradation, compared with non-mannosylated peptide. This, favored antigen presentation and enhanced T-cell activation both *in vitro* and *in vivo* (15).

A second approach that has resulted in improved T-cell activation has been the direct conjugation of long peptides to TLR ligands. TLR-mediated trafficking was described to impact antigen presentation. A study shows that the presence of antigen and TLR ligand in the same endosomes determines entrance to the presentation pathways, suggesting that TLRs or other PRRs might have an important role in determining efficient presentation after antigen uptake (48). Conjugation of antigenic peptides to TLR ligands like the TLR9-ligand CpG or the TLR1/2 heterodimer agonist Pam_3_CSK_4_ have been shown to strongly improve T-cell priming *in vivo* thanks to the combined effect of increased uptake of long peptides and co-delivery with the immune-stimulatory signal (49, 50). Furthermore, the Pam_3_CSK_4_-conjugates were able to establish potent anti-tumor immune responses in multiple preclinical models and are now being tested in a phase I/II clinical trial evaluating synthetic peptide vaccination for treatment of HPV-induced cancers (19, 50) (ClinicalTrials.gov Identifier: NCT02821494). This represents a promising platform for potentiating neo-epitope-based personalized peptide vaccines.

A third targeting strategy includes the conjugation of peptide to a DC-targeting antibody, as reported in a study evaluating the DC-specific receptor DEC205 antibody (51). Targeting viral-specific long peptides to DEC205 promotes peptide uptake by DEC205+ cells and leads to enhanced presentation on MHC class I, which resulted in improved protection to viral challenge. Interestingly, no effect was observed in the efficiency of MHC class II presentation. This highlights the fact that peptide targeting does not only influence which cells will engulf the antigen, but also impacts intracellular trafficking and fate of the antigen for presentation on either MHC class I or II. This becomes more evident in a comparative study on antibody-mediated targeting to either mannose receptor, DEC205, or CD40 in human DCs (52). Targeting of different receptors leads to differential uptake efficiency and endosomal antigen localization. While targeting of the co-stimulatory molecule CD40 was associated to the lowest uptake, it was also associated to the most efficient MHC class II and cross-presentation. In this setting, DEC205-targeting was associated to routing to degradative compartments and low-MHC class I presentation, which could be rescued by inhibiting degradation. These observations expose the complex relations between APC subsets, endosomal routing, and antigen presentation efficiency.

Lastly, an efficient approach is the encapsulation of long peptides in structures such as nanoparticles, liposomes, or nano/hydrogel-systems to enhance T-cell priming by DCs (53–56). Particulate vaccines have been shown to be well internalized by various professional APCs. Properties of these particles, e.g., charge, size, composition, can be modulated to influence uptake by different cells, and vaccine dispersion after injection (57). In the case of liposomes, smaller particles are better internalized by DCs than larger, and positively charged cationic liposomes increase ROS production and cross-presentation (58, 59). The added benefit of nano/hydrogels is the possible incorporation of environmental ques which are slowly released during the induction of DC maturation while peptide can be processed and presented (14). A shared advantage of these delivery systems is the ability to prevent the rapid release of high quantities of free peptide.

## Modulating the Vaccine Response

Recent reports has highlighted that initiation of an adaptive immune response is more than an APC meeting a T cell. Complex interactions of several APC subsets and their crosstalk with other cell types within the vaccination-draining lymph node will determine the outcome of the immune reaction (60–63). Additionally, different APCs can induce different types of immune reactions due to their intrinsic characteristics (61–63). By the application of alternative formulations or conjugations with PRR ligands of peptides-based vaccines, modulation of the immune reaction may be possible by delivering the antigen toward the proper APC to initiate the proper immune response. To optimally design peptide-based vaccines in the future; it is thus necessary to understand the consequences of modifications in APC targeting.

Recently, the importance of cross-presenting DCs in the initiation of an effective anti-tumor immune response was exemplified in several studies (64–66). Tissue originating cross-presenting DCs were shown to be required to migrate from the tumor microenvironment (TME), loaded with antigens from the tumor, toward the draining lymph node, to induce CD8 T cell-dependent delay of tumor outgrowth. This special DC type was characterized by the expression of CD103 and is a DC subtype closely related to the cross-presenting CD8α-expressing DCs that reside in the secondary lymphoid organs (67). These DC subtypes are indicated as part of the type 1 conventional DC (cDC1) group, have a common expression of the previously mentioned C-type lectin receptor DEC205 that was exploited successfully for improved cross-presentation, as well as the “dead cell-receptor” CLEC9A, and has an homolog in the human DC family (68–70). Closely related is the macrophage lineage originating Langerhans cell, which shows similar cross-priming capabilities as cDC1s and is a shared population between mice and humans. Their characteristic expression of c-type lectin receptor Langerin-1 has been used in antibody-mediated targeting to improve cross-presentation and CTL activation (17).

Additionally, the induction of effective CD4 helper responses are crucial for improved CD8 T-cell priming and memory formation, increased tumor infiltration, and local effectiveness (58–60). The DC family has, likewise the conventional DC type 1, a type 2 conventional DC with the characteristic expression of CD11b, which is considered specialized in their capacity to induce T-cell help while lacking CD8 priming capacity (67). However, exploration of specific targeting of this DC subtype for improved helper T-cell priming had negligible attention, due to the intrinsic capacity of most DCs to present in MHC class II. Therefore, it appears more effective to incorporate vaccine modalities which harbor both CD4 and CD8 epitopes and target a wide range of DC subsets, including cross-presenting DCs.

Furthermore, potent peptide-based vaccines can modulate the TME as shown by shifts in the myeloid subpopulations in the tumor (19, 71). Most likely, polarization of CD4 T-cell subsets will regulate the TME to a more proinflammatory state. This is supported by TLR2 ligand-conjugated HPV long peptides which can strongly activate CD4 and CD8 T cells from tumor-draining lymph nodes of cervical cancer patients (50). Other options to modulate the suppressive TME can be achieved by combining cancer vaccines with classical chemotherapy (72) or widely used checkpoint blocking antibodies like anti-PD-1 or anti-PD-L1 (65, 73–78).

The application of TLR-ligand Pam_3_CSK_4_ as targeting moiety was highly promising due to a broad expression of its receptor in dermal DCs (79, 80). The added benefit of a TLR ligand is the combination of a maturation signal with an antigen. Maturation of the DC is known to strongly influence the intracellular machinery and processing of exogenous antigens (81–83). By conjugation of a maturation signal with the antigen, the survival of internalized antigen is increased by the formation of antigen storage depots for prolonged presentation and priming (84). The application of ligands for other PRRs is of interest as well (85). However, care should be taken in using combinations of different PRR ligands in the same modality. Different PRR pathways may affect each other upon simultaneous activation and reduce DC proinflammatory responses, which is exploited by some pathogens (86, 87).

In conclusion, these findings shows that the field is steadily progressing to unravel the relevant cell types involved in optimal (cross-)presentation of antigens. Peptide-based vaccination studies using antigen-bound fluorophores show co-localization with endosomal markers in DC, which correlated with a robust antigen-specific T-cell immune response. However, the strong influence of the relatively large fluorophore on the physico-chemical properties of the antigenic peptide to gain trustworthy physiological information and the limitations to detect peptide intermediates makes interpretations of this complex process difficult. To unravel how activation of APCs orchestrate molecular and cellular mechanisms of antigen processing and presentation operate *in vivo*, and how we can incorporate this knowledge in peptide-based vaccination modalities requires better tracking and visualization tools of vaccine moieties.

## Novel Chemical Visualization Tools

Several technologies to visualize antigens in APCs, other than using T-cell readouts, have been developed in the last decades. Most of these have relied on tracking the activity of an enzyme through a cell. Examples of enzymes used for this are ß-lactamase, luciferase, and horseradish peroxidase. Using these approaches, the endocytic compartments involved in (cross-)presentation could be observed, as well as the cytosolic location of proteins during this event (88–90). Fluorophore-labeled antigens have also been used to study the intracellular movement of antigen in an APC. Using this approach, the presence of intracellular antigen depots was, for example, identified (84).

**Figure 1:**
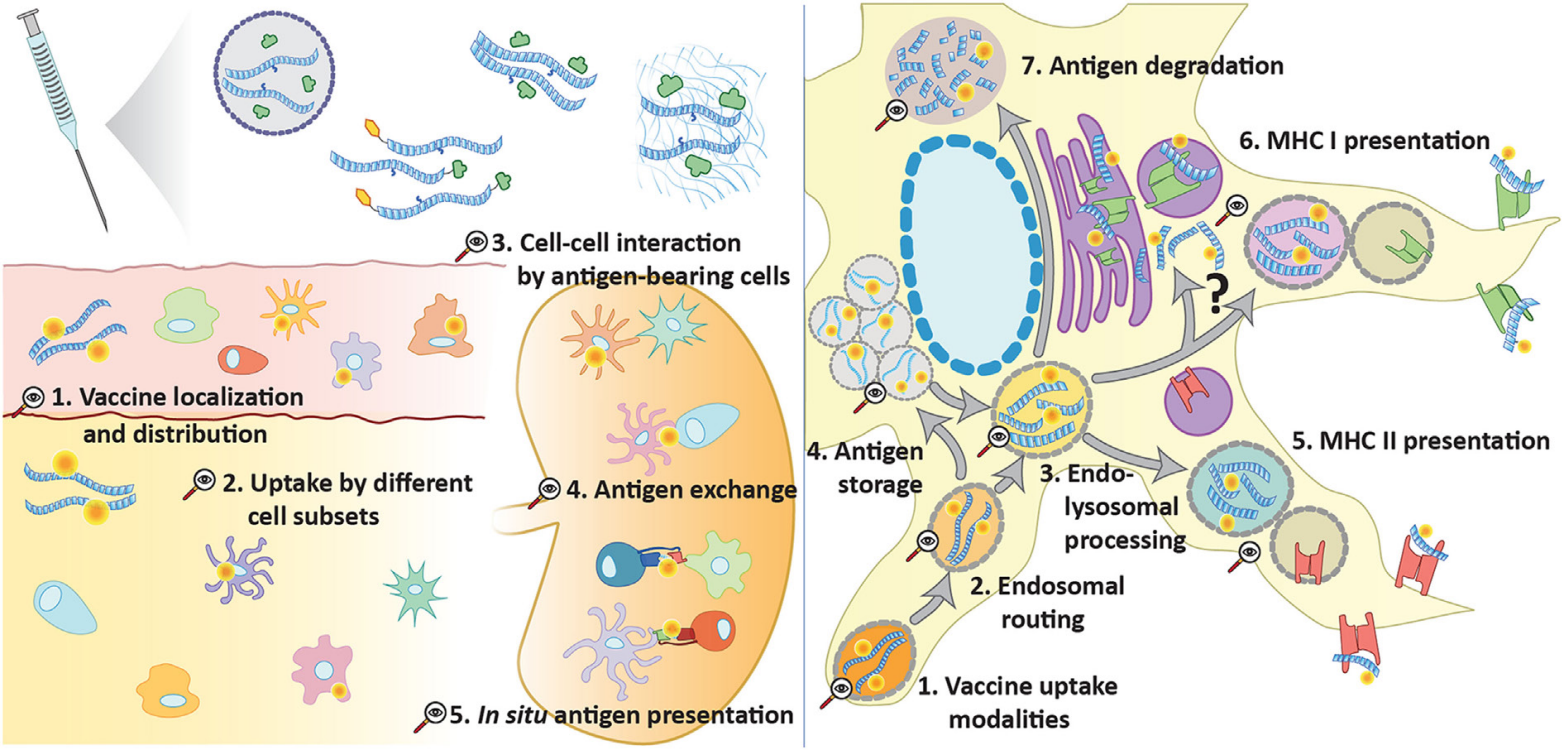
Opportunities for advanced tracking techniques in vaccination. Several forms of peptide antigen can be traced (e.g., *via* click chemistry) at multiple levels to provide further understanding of vaccine processing and induction of adaptive immunity. At a supracellular level, it can unveil vaccine diffusion, drainage, and the main cellular recipients. Tracking of antigen in different cell types can also help to understand the steps involved in the initiation of an immune response, such as cell–cell interaction, antigen exchange, and *in situ* antigen presentation. At a subcellular level, peptide tracking is an important tool to explore the intracellular events that lead to antigen presentation following antigen uptake: endosomal trafficking and sorting to storage compartments, class I or class II presentation or degradation.

However, these approaches also have their constraints. For reporter proteins, the main constraint is that degradation is the hallmark of antigen (cross-)presentation: during antigen presentation any protein must be degraded into peptides to allow for its MHC-loading. As enzyme activities are reliant on largely intact proteins, this means that later stages of the pathway will be invisible using this approach. The use of fluorophore-modified antigens partially solves this by making the detectable signal independent of the intactness of the protein. However, the physicochemical properties of fluorophores must also be considered. Due to their bulky and hydrophobic structure compared with relatively small peptides, fluorophores could strongly influence the behavior of the antigenic peptide and mask epitope residues as well as proteolytic cleavage sites. Moreover, the size of conjugated fluorophores may hamper these peptides to pass through the proteasomal α-annulus of several ångström wide (91). Similarly, peptide translocation by TAP (transporter associated with antigen presentation) to the ER lumen for presentation in MHC class I molecules may be strongly influenced (92). Furthermore, it is difficult, if not impossible, to rule out that constructs lose their fluorophores during processing. As a consequence, not much data exists of later processing stages that could directly visualize antigen. And even in rare cases in which it has been possible [e.g., the H-2K^b^-SIINFEKL pMHC complex antibody 25-D1.16 (93)], translation to other antigens is not obvious. Therefore, a method to thoroughly and accurately apply tracking across a manifold of peptide-based vaccine modalities and the complex cellular interactions involved is highly wanted (see Figure 1).

One field of chemistry of which we are currently exploring the potential is click chemistry (94). This type of chemistry involves a defined ligation reaction between a small *bioorthogonal* chemical group—a chemical group which can be selectively ligated within the context of the living cell or organism—to form a covalent linkage to a detectable group *after* the biological time course has been completed. It is relatively easy (in *Escherichia coli*) to produce bioorthogonally labeled recombinant proteins (95–97) using methionine auxotrophic producer strains in combination with bioorthogonal methionine analogs (98, 99). This chemistry has been applied widely, but its application to immunology is still in its infancy. We ourselves have applied this chemistry to label surface loaded minimal epitopes on the surface of APCs (100) to allow their quantification without using T-cell reagents. However, the reaction is still limited by poor signal-to-noise ratios that cannot compare with the sensitivity of T cells. Detection of the handles in antigens after routing and processing is therefore not yet possible using this approach, despite the groups surviving the antigen presentation pathway (101–103). Once the sensitivity issues can be solved this technique could prove valuable in the imaging of the entire antigen routing pathway with minimal bias. Additionally, this approach may be suited to analyze the *in vivo* fate of chemically defined peptide vaccines. By *ex vivo* secondary staining of relevant cell types or tissues using fluorescent microscopy or histological analysis, the presence and location of the peptide vaccine can be determined. This could be valuable information to improve peptide vaccine design.

## Concluding Remarks

Our current knowledge on innate and adaptive immune system allows us to design molecularly well-defined vaccine moieties. Adjuvant molecules that bind PRR can be synthetically coupled to antigenic peptide sequences. Even though these defined peptide vaccines have strong vaccination capacity, the mechanisms underlying these improvements are only understood to a basal level. To improve the design of peptide-based vaccines, we need to better our understanding of chemically altered vaccines on the events unfolding during vaccination *in vivo*. A major limitation to this understanding is the lack of techniques that allow the study of late stages of antigen processing, and presentation on a cellular and molecular level. Fundamental questions about transfer of peptides within and between cells are currently troublesome since tags or fluorophores are lost and prone to altering essential physicochemical properties due to their bulkiness. The introduction of novel types of chemistry may in future circumvent these problems, which in turn may lead to novel insight in the complex cellular and molecular interactions in immune response induction.

## Author Contributions

BH and ET have contributed equally to the manuscript in writing and figure design. SK and FO have been responsible for scientific input and revisions of the final manuscript.

## Conflict of Interest Statement

The authors declare that the research was conducted in the absence of any commercial or financial relationships that could be construed as a potential conflict of interest.
